# Adherence to World Health Organisation guidelines for treatment of early onset neonatal sepsis in low-income settings; a cohort study in Nepal

**DOI:** 10.1186/s12879-020-05361-4

**Published:** 2020-09-10

**Authors:** Beatrice Ekman, Prajwal Paudel, Omkar Basnet, Ashish KC, Johan Wrammert

**Affiliations:** 1 Pediatric surgery, Children’s hospital, Uppsala, Sweden; 2 Golden Community, Lalitpur, Kathmandu, Nepal; 3 Department of Women’s and Children’s Health, Uppsala University, Uppsala, Sweden

**Keywords:** Neonatal Sepsis, Guideline adherence, Drug resistance, microbial, Socioeconomic factors

## Abstract

**Background:**

Neonatal sepsis is one of the major causes of death during the first month of life and early empirical treatment with injectable antibiotics is a life-saving intervention. Adherence to World Health Organisation guidelines on first line antibiotics is crucial to mitigate the risks of increased antimicrobial resistance. The aim of this paper was to evaluate if treatment of early onset neonatal sepsis in a low-income facility setting observe current guidelines and if compliance is influenced by contextual factors.

**Methods:**

This cohort study used data on antimicrobial treatment of neonatal sepsis onset within 72 h of life from 12 regional hospitals participating in a scale-up trial of a neonatal resuscitation quality improvement package intervention in Nepal. Infants treated according to guidelines were compared with those receiving other antimicrobials. A multiple logistic regression analysis adjusted for the intervention and time trend was applied.

**Results:**

1564 infants with a preliminary diagnosis of early onset sepsis were included. A majority (74.9%) were treated according to guidelines and adherence was increasing over time. Infants born at larger facilities (adjusted Odds Ratio 5.6), those that were inborn (adjusted Odds Ratio 1.97) or belonging to a family of dis-advantaged caste (adjusted Odds Ratio 2.15) had higher odds for treatment according to guidelines. A clinical presentation of lethargy or tachypnoea was associated with adherence to guidelines.

**Conclusion:**

Adherence to guidelines for antibiotic treatment of early neonatal sepsis was moderately high in this low-income setting. Odds for observing guidelines increased with facility size, for inborn infants and if the family belonged to a dis-advantaged caste. Cefotaxime was a common alternative choice when guidelines were not followed, highly relevant for the risk of increased antimicrobial resistance.

**Trial registration:**

ISRCTN, ISRCTN30829654, registered 17th of May, 2017.

## Background

The reduction of neonatal mortality globally continues and road maps like the Every Newborn Action Plan sets ambitious targets and milestones for 2030. Still, deaths during the first month of life continue to increase its proportion of total child mortality before 5 years of age [1]. Invasive newborn infection is one of the major causes of death during the first month of life, especially in high mortality settings [2]. As quality of care and universal health coverage improves world-wide, the availability of injectable antibiotics for infants with sepsis is improving [3]. Empirical treatment of infants presenting with clinical signs of sepsis is a life-saving intervention, but the antimicrobial resistance (AMR) that follow, especially as a result of broad-spectrum antibiotic use, is threatening future progress [4, 5]. It has been estimated that almost one third of the annual 690,000 neonatal deaths from sepsis are associated with AMR [6].

Early onset sepsis (EOS) is defined as bacteraemia in the newborn occurring in the first 72 h of life [7]. Mainly caused by vertical infection from mother to infant, the proportion of EOS compared to late onset sepsis (LOS) has been rapidly decreasing in high-income countries (HIC) because of better Group B Streptococci control during pregnancy [8]. The other common EOS pathogen in HIC is *Escherichia coli*, also commonly infecting vertically from the mother. Studies on pathogen prevalence in low- and middle-income countries (LMIC) are few, but there is some evidence to suggest that Klebsiella species and *Staphylococcus aureus* are more common, indicating horizontal infection [7]. The higher number of potential pathogens in LMIC has led to an increased use of broader spectrum antibiotics for EOS, predominantly in south Asia [6].

Although the panorama of pathogens differs between contexts, there has been no reason to change the World Health Organisation (WHO) guidelines for treatment of neonatal sepsis in health facilities. First choice is still Ampicillin or Benzylpenicillin for 7–10 days with 2 doses of Gentamicin [7]. Also, in an effort to address the burden of AMR, the 20th edition of the *WHO Model List of Essential Medicines* released in 2017 included three groups of antibiotics [9]. ACCESS (affordable and safe antibiotics that should be widely available), WATCH (antibiotics with higher resistance potential recommended as first choice only for a few specific indications or as second choice), and RESERVE (antibiotics that should be restricted for use in specific patients when all other alternatives have failed). The only injectable first choices for EOS in the ACCESS group are Ampicillin and Benzylpenicillin together with Gentamicin as adjuvant.

Apart from the time point of onset before 72 h of age, there is no homogenic definition of EOS in the literature [10]. Blood culture has been used as a golden standard to confirm sepsis but such laboratory evidence is seldom available in LMIC and risks of falsely negative cases has been described [11]. Outside HIC settings, clinicians are generally left to depend only on clinical presentation to detect EOS cases in order to make decisions on empirical antibiotic treatment. WHO has listed symptoms that should be seen as red flags for neonatal sepsis; difficult to feed, lethargy, fast breathing, grunting, sub-costal recessions, fever, hypothermia and central cyanosis. All of those signs could however be present also without an infection, making the case definition difficult [12].

The aim of this paper is to evaluate to what extent treatment of early neonatal sepsis in a low-income facility setting adhere to WHO guidelines and if compliance is influenced by contextual factors or clinical case presentation.

## Methods

Data for this observational cohort study was collected during a large scale-up trial of a neonatal resuscitation quality improvement package in Nepal [13]. The Nepal Perinatal Quality Improvement Project (NePeriQIP) was performed in 12 public second level delivery facilities during 18 months in 2017 and 2018. Data collection for the study presented in this paper stopped when the NePeriQIP ended. The registered trial (ISRCTN30829654) had a stepped-wedge cluster randomized controlled design with a quality improvement intervention targeting quality of care for neonatal resuscitation. The included facilities were of low-volume (> 1000 deliveries a year), medium-volume (> 3000 deliveries a year), and high-volume (> 8000 deliveries a year). One facility from each size group formed one out of totally four wedges (clusters). All facilities provided normal and assisted vaginal delivery along with caesarean section services. Labor units were led by nurse-midwives. For infants with complications, the large and medium sized facilities provided specialised sick newborn care units staffed by pediatricians while small size facilities cared for sick infants at the regular pediatric unit led by medical doctors. The intervention did not contain any education on management of neonatal sepsis. However, all facilities had implemented the *Comprehensive newborn care training package for level II hospital care* introduced by *Department of health services in Nepal* that conform with WHO guidelines for treatment of neonatal sepsis before the start of the study [14].

For this study, infants referred for sick newborn care before 72 h of age with a primary diagnosis of neonatal sepsis, early onset sepsis, pneumonia or acute respiratory infection were included. Antibiotics administered were registered and collected along with data on maternal and infant characteristics. EOS infants treated with Ampicillin or Benzylpenicillin were included in the guideline group and infants treated with all other antibiotics were included in the non-guideline group. Gentamicin was considered an adjuvant treatment used in both groups and thus did not affect the group designation. No follow-up after discharge was performed. An independent data collection team was established at each facility. Data collection was done in paper format and a central research office checked the forms for completeness. A standard operating protocol was applied for data management and quality assurance was done on a quarterly basis.

General, maternal and infant factors were compared for the two groups using Chi-square and Fisher exact tests for categorical variables and student t-tests for continuous variables. Time trend in adherence to guidelines was analysed with linear regression using study month as an independent variable. Odds ratios for significant general characteristics and clinical signs were calculated using univariate logistic regression. A final multiple logistic regression model included all previously significant variables. All logistic regressions were adjusted for time trend and the NePeriQIP intervention. Missing data was excluded from analysis. Stata/IC 16.0 (StataCorp, TX, USA) was used for analysis. Statistical significance was set at *p*-values < 0.05 and confidence intervals (CI) were reported at 95%.

## Results

**Fig. 1:**
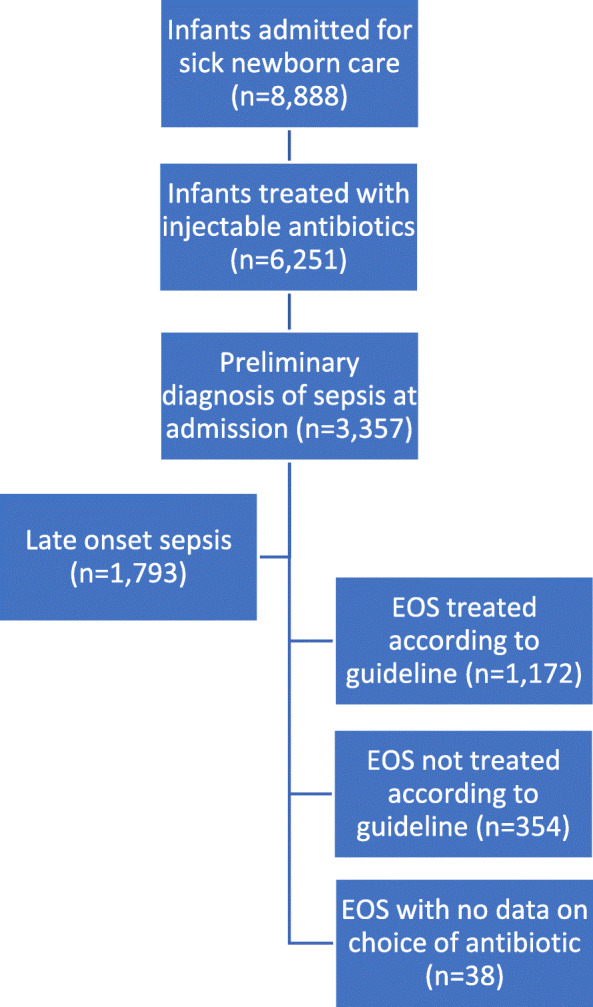
Infants in the Nepal Perinatal Quality Improvement Project included for the study on treatment of early onset sepsis (EOS)

**Fig. 2:**
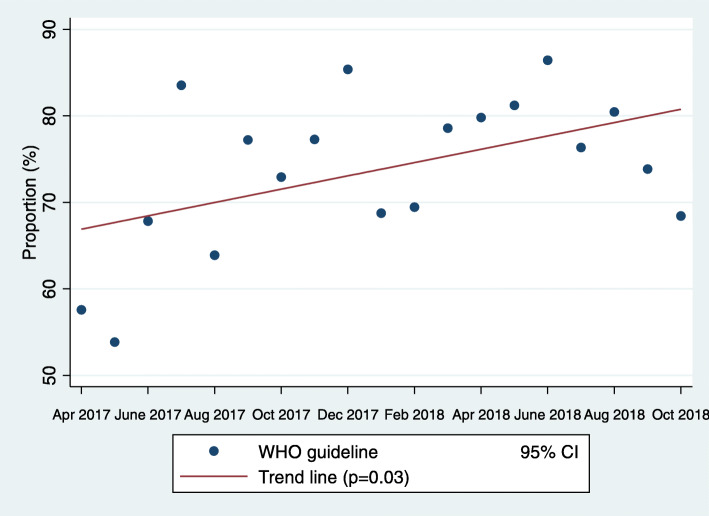
Proportion of infants with early onset sepsis treated according to WHO (World Health Organisation) guidelines over the full study period (18 months)

During the study period, 8888 infants were referred for sick newborn care at the 12 facilities. A majority (70.3%) received injectable antibiotics during care, whereas a little more than one third received a primary diagnosis of sepsis upon admission. Of the sepsis cases, almost half were referred before 72 h of age. Of those EOS cases (*n* = 1564), 74.9% (*n* = 1172) were treated according to WHO guidelines and 22.6% (*n* = 354) were not. For 2.4% (*n* = 38) there was no information on antibiotics available (Fig. 1). The proportion of EOS cases treated according to WHO guidelines varied over time with an increasing trend (*p* = 0.03) during the study (Fig. 2). In the group of patients not treated according to guidelines, Cefotaxime (90%) was the most common antibiotic used, followed by Amikacin (6%), Cloxacillin (2%) and others (2%). Blood culture was only collected in less than 1% of the cases and no results from the cultures were available in the data.

Guideline and non-guideline groups were compared regarding background characteristics. Facility size, the NePeriQIP intervention, inborn infants and belonging to a dis-advantaged caste were associated with treatment according to WHO guidelines. The rate of caesarean section was low in the EOS cohort but did not differ between groups. Death or referral to a higher centre was approximately 10% in the cohort but not different between treatment groups. (Table 1). For clinical signs, lethargy, tachypnoea, grunting, fever, cyanosis and breastfeeding infants were associated with treatment group (Table 2).

**Table 1:** General, maternal and infant factors for infants with early onset sepsis during the NePeriQIP^a^ trial, by antibiotic treatment group; Guideline (*n* = 1172) and Non-Guideline (*n* = 354)

Background characteristic	Guideline n (valid %)	Non-guideline n (valid %)	*p*-value
***General factors***			
Size of hospital			
Large (*n* = 998)	870 (87.2)	128 (12.8)	**< 0.01**
Medium (*n* = 467)	261 (55.9)	206 (44.1)	
Small (*n* = 61)	41 (66.2)	20 (32.8)	
NePeriQIP trial^a^			
Baseline (*n* = 467)	326 (69.8)	141 (30.2)	**< 0.01**
Intervention (*n* = 970)	769 (79.3)	201 (20.7)	
Born at home	62 (6.5)	20 (6.1)	0.81
Inborn infants	719 (82.3)	211 (66.7)	**< 0.01**
***Maternal factors***			
Maternal age in years, mean (SD)	24.5 (4.8)	24.8 (0.27)	0.27
Disadvantaged caste^b^	276 (23.6)	58 (16.4)	**< 0.01**
Caesarean section (elective & emergency)	47 (4.3)	21 (6.1)	0.16
***Infant factors***			
Admission weight, mean (SD)	2820 (612)	2841 (636)	0.58
Gestational age, mean (SD)	38.5 (2.7)	38.9 (2.9)	0.06
Apgar 5 min < 7	53 (8.8)	16 (7.7)	0.64
Outcome (death or referral to higher centre)	96 (8.4)	39 (11.3)	0.10

Missing data: Intervention (*n* = 89); Born at home (*n* = 242); Inborn (*n* = 336); Caesarean section (*n* = 89); Admission weight (*n* = 31); Gestational age (*n* = 616); Apgar 5 min (*n* = 716); Outcome (*n* = 39).

^a^Nepal Perinatal Quality Improvement Project

^b^Dalit and Muslim

**Table 2:** Clinical presentation at the newborn unit for infants with early onset sepsis, by antibiotic treatment group; Guideline (*n* = 1172) and Non-Guideline (*n* = 354)

Characteristics of cases	Guideline n (valid %)	Non-guideline n (valid %)	*p*-value
Lethargy	167 (19.2)	39 (12.3)	**< 0.01**
Tachypnoea (> 60 breaths per minute)	357 (31.7)	61 (17.9)	**< 0.01**
Seizures	41 (4.6)	9 (2.8)	0.18
Grunting	134 (14.8)	13 (4.2)	**< 0.01**
Apnoea	19 (2.1)	2 (0.7)	0.13^a^
Fever (> 38 degrees Celsius)	494 (44.4)	120 (35.5)	**< 0.01**
Hypothermia (< 35.5 degrees Celsius)	9 (0.9)	4 (1.2)	0.75^a^
Severe chest indrawing	27 (3.1)	3 (1.0)	0.06^a^
Central cyanosis	31 (3.5)	3 (0.9)	**0.02**^**a**^
Severe jaundice (palms and soles yellow)	14 (1.66)	4 (1.26)	0.79

^a^Fisher exact test

For variables associated with treatment according to guidelines, crude odds ratios were calculated and adjusted for time trend and the NePeriQIP intervention. There were higher odds of receiving treatment according to guidelines when born at a large facility and lower odds if born at a medium size facility. Infants belonging to a family of dis-advantaged caste or if the infant was inborn had higher odds of guideline treatment. For clinical signs, odds of treatment according to guidelines were higher if the infant presented with lethargy, tachypnoea, grunting or fever (Table 3).

**Table 3:** Crude and adjusted odds for treatment of early neonatal sepsis according to World Health Organisation guidelines

	cOR (95% CI)	aOR^a^ (95% CI)	*p*-value
**Background characteristic**			
Large volume hospital	5.08 (3.95—6.55)	5.03 (3.85—6.60)	**< 0.01**
Medium volume hospital	0.21 (0.16—0.26)	0.21 (0.16—0.28)	**< 0.01**
Small volume hospital	0.61 (0.35—1.05)	0.70 (0.40—1.23)	0.22
Disadvantaged caste^b^	1.57 (1.15—2.15)	1.67 (1.21—2.30)	**< 0.01**
Inborn infant	2.31 (1.73—3.09)	2.24 (1.66—3.03)	**< 0.01**
**Clinical presentation**			
Lethargy	1.69 (1.16—2.46)	1.60 (1.09—2.36)	**0.02**
Tachypnoea (> 60 breaths per minute)	2.12 (1.57—2.89)	1.86 (1.34—2.60)	**< 0.01**
Grunting	3.99 (2.22—7.16)	3.38 (1.87—6.10)	**< 0.01**
Fever (> 38 degrees Celsius)	1.45 (1.13—1.87)	1.42 (1.09—1.85)	**0.01**
Central cyanosis	3.79 (1.15—12.47)	3.32 (1.00—11.02)	0.05

Abbreviations: *cOR* crude odds ratios, *aOR* adjusted odds ratios, *CI* confidence interval

^a^Adjusted for time trend and Nepal Perinatal Quality Improvement Project

^b^Dalit and Muslim

The final model was a multiple logistic regression including all previously significant variables adjusted for time trend and intervention (Table 4). Infants with EOS born at large facilities had more than five-fold higher odds (aOR 5.6, 95% CI 3.75—8.36) of receiving treatment according to guidelines. Odds also increased if the infant was inborn (1.97, 95% CI 1.33—2.93) or if belonging to a family of dis-advantaged caste (aOR 2.15, 95% CI 1.40—3.29). Among clinical signs, odds for receiving treatment according to guidelines was higher if the infant presented with lethargy (aOR 3.22, 95% CI 1.93–5.38) or tachypnoea (aOR 1.96, 95% CI 1.21—3.17).

**Table 4:** Multiple logistic regression of background characteristics and clinical signs with odds for treatment of early neonatal sepsis according to World Health Organisation guidelines

Variable	Adjusted odds ratio^a^ (95% CI)	*p*-value
Large hospital (small and medium as reference)	5.60 (3.75—8.36)	**< 0.01**
Disadvantaged caste (Dalit and Muslim)	2.15 (1.40—3.29)	**< 0.01**
Inborn (infant not referred from other facility)	1.97 (1.33—2.93)	**< 0.01**
Lethargy (decreased level of consciousness)	3.22 (1.93—5.38)	**< 0.01**
Tachypnoea (> 60 breaths per minute)	1.96 (1.21—3.17)	**< 0.01**
Grunting	1.24 (0.53—2.91)	0.63
Fever (temperature over 38 degrees Celsius)	0.70 (0.48—1.04)	0.08

^a^Adjusted for time trend and Nepal Perinatal Quality Improvement Project

## Discussion

In this study of almost 9000 infants admitted to sick newborn care units at twelve regional delivery facilities in Nepal we found a high utilisation of antibacterial treatment. More than two thirds of all infants at the units received injectable antibiotics, suggesting a high rate of prophylactical application in addition to treatment of suspected sepsis. This high prevalence is in line with previous data and probably associated with the high rate of nosocomial infection in this and comparable contexts [7]. In a web-based survey among hospitalized children and neonates from eight countries in Asia, it was found that 88% of patients received at least one parenteral antibiotic [15]. This potential overuse of antibiotics is however global. In HIC it has been estimated that up to 50% of all antimicrobial prescriptions in neonatal intensive care units (NICU) are inappropriate [16].

Focusing on infants with EOS, we have demonstrated that almost three quarters were treated according to WHO guidelines using first line Ampicillin or Benzylpenicillin with or without Gentamicin. This moderately high adherence to guidelines was rising during the study period, indicating an increased awareness of the national protocol for newborn care but also of the risks from AMR among the clinicians working at the facilities. In Nepal, AMR has previously been somehow neglected because of other pressing public health priorities [17]. As a response, the Ministry of Health and Population in 2014 issued guidelines for national antibiotic treatment in the healthcare sector [18]. This could have gradually affected knowledge of AMR in Nepal and explain the change in treatment according to guidelines over the course of the study.

Larger facilities had better adherence to guidelines. There is no data on antibiotic prescription variation over health facilities in Nepal, but we know from research in HIC that the behaviour of physicians can vary a lot in, and between, countries when it comes to utilisation and prescription of antibiotics to children [19]. Delivery volume can also be expected to correlate with the size of the faculty or other leadership entities that influence, and enforce, local guidelines. A study of 127 NICU:s in the US reported that community units had higher variation in antibiotic use rate than regional ones, although the burden of proven infection was similar [20]. This greater variation of use suggests that the choice of antibiotics could be related to the behaviour of a prescribing physician rather than to guidelines, especially in smaller units.

For infants not treated with first-line antimicrobials, Cefotaxime was most commonly used. For some time, it has been known that Cefotaxime and other third generation cephalosporins are particularly strong drivers of AMR. Already in 2000, a study performed in a Netherland NICU found an 18-fold higher rate of colonisation with strains resistant to empiric therapy when infants were treated with Cefotaxime compared to the alternative regimen of Benzylpenicillin [21]. Also, there is evidence that resistance to Cefotaxime is alarmingly high in south Asian contexts [22]. Cefotaxime is also more expensive. The cost for treatment of a 5 kg neonate during a 7 days course is up to five times higher than recommended first line antibiotics for neonatal sepsis [7]. In our study including 1564 infants with EOS, blood culture was available only in 15 cases. This supports the conclusion that the moderately frequent use of Cefotaxime is also empirical and not guided by microbial findings. There was a trend of higher mortality or referral to a higher centre in the group not treated according to guidelines in our data. The data on mortality in our study should be interpreted with caution as it only includes in-hospital deaths. We had no information on neonatal deaths after discharge as there was no follow-up performed. However, this finding may be explained by physician’s choice to use broader antibiotics for more severe clinical cases. This strategy is not supported by evidence, in a recent study from the US, mortality was higher in infants empirically treated with Cefotaxime even after adjusting for confounding factors [23]. Given the resistance pattern, the potential of increased AMR, the risk of worse outcomes and also cost, empirical use of Cefotaxime should be discouraged and its use restricted to cases guided by blood culture or in patients were first-line treatment fails [9].

Dis-advantaged caste was associated with the choice of antibiotics for EOS cases. It is worrisome that odds for treatment according guidelines was higher in this group despite that the data was from government funded, free-of-charge, facilities. In LMIC, it is common that additional, more advanced, or better quality of care, is offered at government facilities if families pay extra [24]. There is also evidence to support that patient sociodemographic status can affect decisions on antibiotic treatment taken by healthcare staff [25]. The result in this study indicates that out-of-pocket expenditure could lead to a higher use of broader spectrum antibiotics perceived as better or safer, resulting in higher AMR pressure without evidence for better outcomes. Inborn infants also had higher odds for treatment according to guidelines, suggesting that referred infants were to a higher extent treated with second line antibiotics such as Cefotaxime. Physicians might be more vigilant towards infants referred from other facilities but for first-line empirical treatment of EOS within 72 h after birth, out-born infants should receive the same antibiotic regimen as inborn peers.

There has been attempts to stratify infants in HIC settings into risk groups according to clinical signs combined with maternal risk factors. The intention has been to guide the choice between empirical treatment or a wait and observe approach to reduce the total utilisation of antimicrobials [26]. Several clinical red flags for EOS were associated with antibiotic choice in the crude logistic regression. The final model found that patients with lethargy and tachypnoea had higher odds for adherence to WHO guidelines. A plausible explanation could be that when physicians encounter a case that clinically presents as sepsis, they observe guidelines to a higher extent [12]. Consequently, in more ambiguous cases, a broader second line treatment such as Cefotaxime is chosen. As we have argued above, this is not supported by evidence for EOS cases.

This study has some weaknesses. Firstly, in the Nepali protocol used for second level government facilities, Cefotaxime is recommended for meningitis in newborn infants. In our data, few infants (*n* = 3) were reported as meningitis cases and they were not included in the cohort. This low incidence however suggest that meningitis cases have instead received a sepsis diagnose. This could potentially underestimate the rate of treatment according to protocol in our study. Secondly, there could be a reporting bias, as there was missing data on antibiotic treatment and those cases were excluded from the study. Thirdly, the data only included the initial assessment and antibiotic choice. We could not distinguish between infants initially treated with first-line antibiotics were other antibiotics were added later because of blood culture reports or patient deterioration. Finally, there could also be some cases where Cefotaxime was used as an adjuvant to Ampicillin or Benzylpenicillin, as suggested by some guidelines used outside Nepal [7]. This is however unlikely, as Gentamicin according to the data was widely available in all facilities over the course of the study.

The data in this study was collected from 12 delivery facilities across Nepal. Facilities differed in size, geographical location, number of staffs, and experience of the medical staff. The cohort was relatively large with more than 1500 EOS cases over a period of 18 months. No other data than background characteristics and clinical presentation and management was collected. This is often the case in low-income situated settings, where lab data or other diagnostics are usually not available, at least outside the tertiary settings. The facts above allow for possible generalisation of the results to other comparable settings. In low-income countries, it is common to use recommendations by WHO in national clinical protocols. However, protocols may be subject to national traditions and adherence to antimicrobial recommendations depend on many other factors as discussed above. Therefore, although factors found influencing the choice of antibiotic treatment has an external validity, our results of a moderately high adherence to WHO recommendations in this setting should be used with caution for other low-income countries.

## Conclusion

Adherence to World Health Organisation guidelines for antibiotic treatment of early neonatal sepsis was moderately high and improving over time in this low-income situated sick newborn care setting. Inborn infants, infants from families with low socio-economic status and delivered at high-volume facilities were more likely to be treated according to guidelines. Clinical case presentation influenced antibiotic choice in this study but as EOS treatment is empirical and not initially guided by blood culture, first choice should always follow guidelines. Cefotaxime was common in infants not treated according to guidelines, but it should be restricted to confirmed sensitive cases or as second line treatment. Raised awareness of risks from antimicrobial resistance and interventions to increase adherence to guidelines is needed, especially in facilities with lower delivery volume.

## Data Availability

The datasets used and/or analysed during the current study are available from the corresponding author on reasonable request.

## Abbreviations

AMR: Antimicrobial resistance
aOR: Adjusted odds ratio
CI: Confidence interval
EOS: Early onset sepsis
HIC: High-income country
LMIC: Low- and middle-income country
LOS: Late onset sepsis
NePeriQIP: Nepal perinatal quality improvement project
NICU: Neonatal intensive care unit
OR: Odds ratio
WHO: World health organisation
